# Predictors of loss to follow-up from HIV antiretroviral therapy in Namibia

**DOI:** 10.1371/journal.pone.0266438

**Published:** 2022-04-14

**Authors:** Steven Y. Hong, Anna Winston, Nicholus Mutenda, Ndapewa Hamunime, Tuhin Roy, Christine Wanke, Alice M. Tang, Michael R. Jordan

**Affiliations:** 1 Division of Geographic Medicine and Infectious Diseases, Tufts Medical Center, Boston, Massachusetts, United States of America; 2 Department of Public Health and Community Medicine, Tufts University School of Medicine, Boston, Massachusetts, United States of America; 3 Hospital of the University of Pennsylvania, Philadelphia, Pennsylvania, United States of America; 4 Directorate of Special Programmes, Republic of Namibia Ministry of Health and Social Services, Windhoek, Namibia; National Institute for Communicable Disease (NICD), South Africa, SOUTH AFRICA

## Abstract

Despite progress on population-level HIV viral suppression, unknown outcomes amongst people who have initiated antiretroviral therapy (ART) in low- and middle-income countries, commonly referred to as loss to follow-up (LTFU), remains a barrier. The mean global estimate of LTFU is 20%, exceeding the World Health Organization target of <15%. Pervasive predictors associated with LTFU include younger age, low body mass index, low CD4 count, advanced HIV clinical stage and certain ART regimens. In Namibia, ART use by eligible individuals exceeds 85%, surpassing the global average. Nonetheless, LTFU remains a barrier to achieving viral suppression and requires research to elucidate context-specific factors. An observational cohort study was conducted in Namibia in 2012 by administering surveys to individuals who presented for HIV care and initiated ART for the first time. Additional data were collected from routine medical data monitoring systems. Participants classified as LTFU at 12 months were traced to confirm their status. Predictors of LTFU were analyzed using multivariable logistic regression. Of those who presented consecutively to initiate ART, 524 were identified as eligible to enroll in the study, 497 enrolled, and 474 completed the baseline questionnaire. The cohort had mean age 36 years, 39% were male, mean CD4 cell count 222 cells/mm3, 17% were WHO HIV clinical stage III-IV, and 14% started efavirenz-based regimens. Tracing participants classified as LTFU yielded a re-categorization from 27.8% (n = 132) to 14.3% (n = 68) LTFU. In the final multivariable model, factors associated with confirmed LTFU status were: younger age (OR 0.97, 95% CI 1.00–1.06, p = 0.02); male sex (OR 2.34, CI 1.34–4.06, p = 0.003); difficulty leaving work or home to attend clinic (OR 2.55, CI 1.40–4.65, p = 0.002); and baseline efavirenz-based regimen (OR 2.35, CI 1.22–4.51, p = 0.01). Interventions to reduce LTFU should therefore target young men, particularly those who report difficulty leaving work or home to attend clinic and are on an efavirenz-based regimen.

## Introduction

Tremendous progress has been made in combating the global HIV pandemic, as represented by progress towards the UNAIDS 90-90-90 targets. This equates to a target of at least 73% of people living with HIV (PLHIV) achieving viral suppression. Namibia was one of 14 countries to have achieved this goal [1]. Per the global average, 59% of PLHIV were virally suppressed in that 81% of PLHIV knew their HIV status, of which 83% were on antiretroviral therapy (ART), of which 88% were virally suppressed [1].

Global efforts aimed at increasing ART coverage (meaning increased eligibility criteria, availability and prescribing to culminate in increased ART use) has been imperative to working to achieve the global 90-90-90 goals. However, increased global ART coverage has been accompanied by barriers to retention on ART, loss to follow-up (LTFU), and drug resistance–thereby jeopardizing the goal of viral suppression [2,3]. As global ART coverage increased from 7% in 2005 to 62% in 2018, the global LTFU rate 12 months after ART initiation increased from 11.9% in 2004 to 24.5% in 2012 (with only a marginal drop to 20% in 2016) [2,4]. In resource-limited settings, an average of 25% of patients are LTFU by 12 months, exceeding the goal of <15% set by the WHO [2,5–8]. Over the same time period since ART coverage began increasing in 2001, the prevalence of drug resistant HIV tripled from 11% to 29% from 2001 to 2016 [9].

In Namibia, ART coverage surpassed the global average by increasing from 8% in 2005 to 85% in 2019 [10]. However, challenges remain with treatment interruption, drug resistance and viral suppression [11]. For example, a prior study showed that 20.8% of all ART starters had at least a two-month period of absence from their ART site during the first year of treatment, which can lead to treatment interruptions and drug resistance [12]. A small sub-sample of a 2017 study demonstrated 25% non-nucleoside reverse transcriptase inhibitor (NNRTI) resistance [11]. An NNRTI was part a first-line ART regimen until recently when the WHO replaced this with the integrase inhibitor dolutegravir (DTG) [13]. DTG has evidence that it is more effective, easier to take, and has fewer side effects and less risk of resistance [13].

Transition to DTG-containing first-line ART is currently underway in 82 low- and middle-income countries, including Namibia. Despite the benefits of DTG, adherence is still paramount and will still require further identification of predictors of LTFU to retain individuals in care. Pervasive predictors prior associated with ART LTFU include: younger age, low body mass index (BMI), low CD4 counts at treatment initiation, advanced HIV clinical stage and select ART regimens, among other factors cited less frequently [14–20]. It is important to not only identify demographic and clinical characteristics, but also social risks and barriers to adherence to optimally offer interventions.

## Materials and methods

An observational cohort study of individuals who initiated ART for the first time at one of the seven participating ART sites was conducted to assess predictors of LTFU. The seven participating ART sites were randomly selected from the 36 eligible public ART sites across six geographic regions in Namibia. Sites eligible for inclusion had 1) a LTFU rate >15%, 2) started at least 134 participants on ART per year, and 3) not previously intensified participant tracing to a level greater than standard of care. Participant inclusion criteria included age at least 18 years and standard clinical eligibility to initiate a first-line adult ART regimen. Exclusion criteria included previous initiation of ART (including if the participant was currently on ART or had stopped). Recruitment included offering study participation to all patients meeting eligibility criteria who presented consecutively to one of the participating public ART sites to initiate ART until the enrollment goal was met. Recruitment occurred from August 2012 to October 2012. This population can be considered representative of the general population given the ART sites were randomly selected across the nation as participants were selected randomly based on consecutive presentation to these sites.

Written informed consent was obtained from participants at enrollment to 1) complete a baseline questionnaire separate from routine care, 2) access routinely collected medical and pharmacy records for ART program monitoring, and 3) be traced at 12 months. The baseline questionnaire included multiple choice and short-answer questions about demographics, socioeconomic status, nutrition and health status, beliefs about healthcare, barriers to healthcare access, and HIV-related stigma (S1 File) [16]. All questionnaires were translated from English into Afrikaans and Oshiwambo (together representing >95% of Namibian participants’ language capabilities), back-translated, and pre-tested with local staff. No formal validation was performed. The questionnaire included the Household Food Insecurity Access Survey (HFIAS) and a Depression Screener (PHQ-9) [21,22]. Clinical data were abstracted from Electronic Patient Management System and the Electronic Dispensing Tool by running existing automated queries in collaboration with the Ministry of Health and Social Services (MoHSS).

LTFU was assessed based on the routine medical and pharmacy records. Participants were initially classified by the World Health Organization (WHO) definition of LTFU. At the time of this study, the WHO defined patients as LTFU if they had not returned to clinic within 90 days of a missed appointment at the 12-month date after ART initiation and were not deceased nor transferred (meaning they were excluded from the LTFU cohort if they were active, deceased or transferred to a different clinic). Since analysis was completed, the WHO definition of LTFU was updated to a 28-day lapse since the last missed appointment and thus the new definition was not incorporated into analysis [23]. For all participants who were classified as LTFU, phone and physical tracing were attempted to confirm an accurate status. Characteristics of participants engaged in care at 12 months were compared with those of participants classified as LTFU to assess predictors of LTFU by utilizing the baseline questionnaire and data abstracted from medical and pharmacy records.

Characteristics of participants who were LTFU were compared to those engaged in care using the χ2 or Fisher’s exact test for categorical variables, and the student t-test or Wilcoxon rank sum test for continuous variables. Variables with initial hypothesis testing p<0.05 were included in univariable logistic regression. Univariable logistic regression models were fit for each potential predictor with LTFU (yes/no) as the outcome. All predictors that were associated with LTFU in the univariable models (p<0.20) were entered into a multivariable logistic regression model. A backwards selection process was used, dropping non-significant (p>0.05) predictors one by one, until the final model was reached. As each predictor was dropped from the model, it was checked to make sure that removing the variable did not affect the other coefficients in the model. Time to event analysis was also conducted.

Approval was obtained from the Namibia MoHSS Ethics and Research Committee and the Tufts Medical Center and Tufts University School of Medicine Institutional Review Board. Participating in this study posed minimal risk to participants. Appropriate measures were taken to keep all research data confidential.

## Results

### Cohort description

A total of 524 individuals of those who presented to initiate ART were identified as eligible to enroll in the study, 497 enrolled, and 474 completed the baseline questionnaire. Of the 474 participants included in analyses, 27.8% (n = 132) were LTFU prior to tracing, compared to 14.3% (n = 68) after tracing. The overall mean age of participants was 36.1 years old, and the majority were female (61.2%), not employed (53.5%), and had monthly household income below N$500 (58.82 USD in 2012) (Table 1). The mean baseline CD4 count was 222 cells/mm^3^, and most had baseline WHO HIV Stage I-II (83.2%). Most participants were prescribed a nevirapine-based baseline ART regimen (86.3%), while the remainder were prescribed an efavirenz-based regimen (13.7%).

**Table 1:** Cohort characteristics overall and stratified into alive and active on ART versus LTFU 12 months after ART initiation.

Characteristic	Overall	Active on ART	LTFU		
	Mean (±SD) or N (%)	Mean (±SD) or N (%)	Mean (±SD) or N (%)	P-value	Test
	N = 474 (100%)	N = 406 (85.7%)	N = 68 (14.3%)		
**Demographics**					
**Age (years)**	36.1 (±10.7)	36.4 (±10.9)	34.1 (±9.2)	0.108	t-test
**Sex**				0.003	χ2
Male	182 (38.8%)	145 (36.1%)	37 (55.2%)		
Female	290 (61.2%)	257 (63.9%)	30 (44.2%)		
**Language**				<0.001	Fisher’s exact
English	135 (28.5%)	104 (25.6%)	31 (45.6%)		
Oshiwambo	262 (55.3%)	243 (59.9%)	19 (27.9%)		
Afrikaans	44 (9.3%)	31 (7.6%)	13 (19.1%)		
Silozi	33 (7.0%)	28 (6.9%)	5 (7.4%)		
**Religion**				0.035	χ2
Christian (excluding Pentecostal)	363 (76.6%)	317 (78.1%)	46 (67.7%)		
Pentecostal	93 (19.6%)	77 (19.0%)	16 (23.5%)		
Traditional, other, none	18 (3.8%)	12 (3.0%)	6 (8.8%)		
**Education**				0.186	Fisher’s exact
None or primary	191 (40.4%)	157 (38.8%)	34 (50.0%)		
Secondary or above	282 (59.6%)	248 (61.2%)	34 (50.0%)		
**Not employed**				0.07	χ2
Yes	252 (53.5%)	223 (55.2%)	29 (43.3%)		
No	219 (46.5%)	181 (44.8%)	38 (56.7%)		
**Average household size**	6.3 (±4.5)	6.4 (±4.6)	5.5 (±3.7)	0.121	t-test
**Monthly household income**				0.121	Fisher’s exact
N$ 0–500 (0–59 USD)	235 (58.9%)	205 (61.2%)	30 (46.9%)		
N$501–1000 (59–118 USD)	65 (16.3%)	49 (14.6%)	16 (25.0%)		
N$ 1001–2500 (118–294 USD)	64 (16.0%)	54 (16.1%)	10 (15.6%)		
N$ 2501–5000 (284–588 USD)	21 (5.3%)	16 (4.8%)	5 (7.8%)		
N$ 5000+ (588+ USD)	14 (3.5%)	11 (3.3%)	3 (4.7%)		
**Marital status**				0.744	Fisher’s exact
Married, living together	189 (40.0%)	158 (39.0%)	31 (45.6%)		
Divorced, separated, widowed	52 (11.0%)	45 (11.1%)	7 (10.3%)		
Never married, never lived together	232 (49.1%)	202 (49.9%)	30 (44.1%)		
**Nutrition**					
**Food insecurity** ^a^				0.098	Fisher’s exact
Secure to mild insecurity	332 (70.5%)	293 (72.5%)	39 (58.2%)		
Severe to moderate insecurity	139 (29.5%)	111 (27.5%)	28 (41.8%)		
**Baseline weight (kg)**	59.2 (±11.3)	59.6 (±11.2)	56.9 (±11.6)	0.079	t-test
**Health status**					
**Baseline CD4, cells/mm** ^**3**^	222 (±117)	224 (±114)	212 (±117)	0.467	t-test
**Baseline WHO HIV clinical stage** ^b^				0.011	Fisher’s exact
I or II	385 (83.2%)	341 (85.5%)	44 (68.8%)		
III or IV	78 (16.9%)	58 (14.5%)	20 (31.3%)		
**Baseline ART regimen**				0.003	χ2
Nevirapine-based	409 (86.3%)	358 (88.2%)	51 (75.0%)		
Efavirenz-based	65 (13.7%)	48 (11.8%)	17 (25.0%)		
**Self-reported health rating**				0.299	χ2
Excellent, very good	59 (12.5%)	48 (11.9%)	11 (16.4%)		
Good, fair, poor	412 (87.5%)	356 (88.1%)	56 (83.6%)		
**Depression screen (PHQ-9 score)** ^c^				0.722	Fisher’s exact
Minimal to mild (0–5)	379 (81.9%)	328 (82.4%)	51 (78.5%)		
Moderate to moderately severe (6–20)	84 (18.1%)	70 (17.6%)	14 (21.5%)		
**Health beliefs**					
**How important do you think it is to see a doctor routinely for your HIV care?**				1	Fisher’s exact
Very important	435 (92.0%)	372 (91.9%)	63 (92.7%)		
Somewhat important	28 (5.9%)	24 (5.9%)	4 (5.9%)		
Not important	10 (2.1%)	9 (2.2%)	1 (1.5%)		
**Religious leader told to pray instead of ART**				0.056	χ2
Yes	21 (4.5%)	15 (3.7%)	6 (9.0%)		
No	447 (95.5%)	386 (96.3%)	61 (91.0%)		
**Prefer traditional medicine**				0.002	Fisher’s exact
Yes	5 (1.1%)	1 (0.3%)	4 (6.0%)		
No	456 (98.9%)	393 (99.8%)	63 (94.0%)		
**Healthcare access barriers**					
**Difficulty to leave work or home to attend clinic**				0.003	χ2
Easy to little difficult	387 (81.8%)	340 (84.0%)	47 (69.1%)		
Difficult to very difficult	86 (18.2%)	65 (16.1%)	21 (30.9%)		
**Difficulty traveling to clinic**				0.018	χ2
Easy to little difficult	385 (81.9%)	337 (83.6%)	48 (71.6%)		
Difficult to very difficult	85 (18.1%)	66 (16.4%)	19 (28.4%)		
**Average travel time (minutes)**	55.4 (±83.4)	55 (±86.5)	57.7 (±61.9)	0.806	t-test
**Average travel cost (N$)**	19.9 (±23.3)	20.5 (±23.3)	16.6 (±23.1)	0.241	t-test
**Walk to clinic**				0.332	χ2
Yes	129 (27.9%)	107 (27.1%)	22 (32.8%)		
No	333 (72.1%)	288 (72.9%)	45 (67.2%)		
**Taxi/bus**				0.912	χ2
Yes	149 (32.3%)	127 (32.2%)	22 (32.8%)		
No	313 (67.8%)	268 (67.9%)	45 (67.2%)		
**Hitchhike**				0.022	χ2
Yes	153 (33.1%)	139 (35.2%)	14 (20.9%)		
No	309 (66.9%)	256 (64.8%)	53 (79.1%)		
**Months lived in baseline town during study period**				0.049	χ2
12	410 (86.9%)	356 (88.1%)	54 (79.4%)		
<12	62 (13.1%)	48 (11.9%)	14 (20.6%)		
**HIV stigma**					
**Family/friends support ARTs**				0.305	Fisher’s exact
Supportive, very supportive	419 (88.4%)	361 (88.9%)	58 (85.3%)		
Neutral	40 (8.5%)	34 (8.4%)	6 (8.8%)		
Opposed, very opposed	13 (2.7%)	9 (2.2%)	4 (5.9%)		
**Stigma score** ^d^				0.022	χ2
0–1	260 (56.2%)	232 (58.3%)	28 (43.1%)		
2–6	203 (43.8%)	166 (41.7%)	37 (56.9%)		
**Non-disclosure of HIV status to certain persons**				0.085	χ2
Yes	250 (53.0%)	208 (51.4%)	42 (62.7%)		
No	222 (47.0%)	197 (48.6%)	25 (37.3%)		

^a^Food insecurity: Household Food Insecurity Access Survey [21].

^b^Baseline WHO HIV clinical stage.

^c^Depression screen: PHQ-9 [22].

^d^Stigma score: Stigma questionnaire developed for study.

Most participants thought that seeing a doctor routinely for HIV care was very important (92%), felt that family and friends were supportive of ARTs (88.4%), and reported a low stigma score (56.2%). However, over half (53%) still reported non-disclosure of their HIV status to certain persons. A minority (4.5%) had been advised to pray instead of take ARTs by a religious leader, and only 1.1% reported preferring traditional medicine. Most participants (81.8%) felt it was easy or a little difficult to leave work or home to attend an ART clinic, as most participants (81.9%) felt it was easy or a little difficult to travel to clinic despite an average travel time of almost one hour. The most common modes of transportation to clinic were hitchhiking (33.1%), taxi/bus (32.3%), and walking (27.9%).

### Univariable analysis

In the univariable model, factors associated with increased LTFU included being male, religion as traditional/other/none, food insecurity, baseline WHO stage III-IV, baseline efavirenz-based regimen, preference for traditional medicine, difficulty leaving home or work to get to clinic, difficulty traveling to clinic, hitchhiking, and stigma (Table 2). Factors associated with decreased LTFU included language as Oshiwambo and spending all 12 months in the town of baseline questionnaire.

**Table 2:** Characteristics associated with LTFU in univariable and multivariable analysis.

	Univariable			Multivariable		
Characteristic	OR	(95% CI)	P-value	OR	(95% CI)	P-value
**Demographics**						
**Age**	0.98	(0.95, 1.00)	0.11	0.97	(1.00, 1.06)	0.02
**Sex**						
Male	2.19	(1.30, 3.69)	0.003	2.34	(1.34, 4.06)	0.003
Female	Ref			Ref		
**Language**						
English	Ref					
Oshiwambo	0.26	(0.14, 0.49)	<0.001			
Afrikaans	1.41	(0.66, 3.01)	0.38			
Silozi	0.60	(0.21, 1.68)	0.33			
**Religion**						
Christian non-Pentecostal	0.70	(0.38, 1.30)	0.26			
Pentecostal	Ref					
Traditional, other, none	2.41	(0.79, 7.36)	0.05			
**Nutrition**						
**Food insecurity** ^a^						
Secure to mild insecurity	Ref					
Moderate to severe insecurity	1.90	(1.11, 3.23)	0.02			
**Health status**						
**Baseline WHO HIV clinical stage** ^b^						
I or II	Ref					
III or IV	2.67	(1.47, 4.86)	0.001			
**Baseline ART regimen**						
Nevirapine-based	Ref			Ref		
Efavirenz-based	2.49	(1.33, 4.65)	0.003	2.35	(1.22, 4.51)	0.01
**Health beliefs**						
**Prefer traditional medicine**						
Yes	24.95	(2.74, 226.9)	0.004			
No	Ref					
**Healthcare access barriers**						
**Difficulty to leave work or home to attend clinic**						
Easy to little difficult	Ref			Ref		
Difficult to very difficult	2.34	(1.31, 4.17)	0.004	2.55	(1.40, 4.65)	0.002
**Difficulty traveling to clinic**						
Easy to little difficult	Ref					
Difficult to very difficult	2.02	(1.12, 3.66)	0.02			
**Hitchhiked to clinic**						
Yes	Ref					
No	2.06	(1.10, 3.84)	0.02			
**Months lived in baseline town during study period**						
12	0.52	(0.27, 1.01)	0.05			
<12	Ref					
**HIV stigma**						
**Stigma score** ^c^						
0–1	Ref					
2–6	1.85	(1.09, 3.14)	0.02			

^a^Food insecurity: Household Food Insecurity Access Survey [21].

^b^Baseline WHO HIV clinical stage.

^c^Stigma score: Stigma questionnaire developed for study.

### Multivariable analysis

In the multivariable model, factors that remained independently associated with LTFU were younger age (OR 0.97, CI 1.00–1.06, p = 0.02), male sex (OR 2.34, CI 1.34–4.06, p = 0.003), difficulty leaving work or home to attend clinic (OR 2.55, CI 1.40–4.65, p = 0.002), and baseline efavirenz-based regimen (OR 2.35, CI 1.22–4.51, p = 0.01) (Table 2). This multivariable model was built using backwards selection process, and the remainder of variables were not significant. Variables that were significant in the univariable model but dropped out of the multivariable model included language, religion, food insecurity, baseline WHO HIV clinical stage, preference for traditional medicine, difficulty traveling to clinic, hitchhiking to clinic, months lived in baseline town during study period, and stigma score. Time to event analysis was also conducted and yielded no difference in results, thus the original analysis method was used.

### Pre- versus post-tracing LTFU cohorts

Of the 132 patients who were LTFU prior to tracing, half (n = 66) remained LTFU after tracing, representing a drop from 28% of the total cohort to 14% LTFU. (Note that 68 instead of 66 participants were included in post-tracing LTFU analysis above because in addition to the 66 patients who remained LTFU after tracing, two patients who were initially active were also found to be LTFU post-tracing.) Of those with the final classification of LTFU, their pre-tracing classifications per clinical and/or pharmacy records were LTFU or unknown (n = 54), dead (n = 8), silent transfer (n = 4), or active (n = 2). Factors that were significantly associated with unsuccessful tracing (or remaining LTFU post-tracing) included language, baseline WHO HIV clinical stage, average travel cost, hitchhiking to clinic, and stigma score (Table 3).

**Table 3:** Characteristics of patients pre-tracing LTFU and stratified into post-tracing active versus post-tracing LTFU 12 months after ART initiation.

Characteristic	Overall	Post-tracing active	Post-tracing LTFU		
	Mean (±SD) or N (%)	Mean (±SD) or N (%)	Mean (±SD) or N (%)	P-value	Test
	N = 132 (100%)	N = 66 (50%)	N = 66 (50%)		
**Demographics**					
**Age (years)**	34.3 (±11.5)	34.6 (±13.3)	34.1 (±9.3)	0.797	t-test
**Sex**				0.114	χ2
Male	61 (46.9%)	26 (40.0%)	35 (53.9%)		
Female	69 (53.1%)	39 (60.0%)	30 (46.2%)		
**Language**				0.004	Fisher’s exact
English	51 (38.6%)	30 (31.8%)	30 (45.4%)		
Oshiwambo	55 (41.7%)	37 (56.1%)	18 (27.3%)		
Afrikaans	17 (12.9%)	4 (6.1%)	13 (19.7%)		
Silozi	9 (6.8%)	4 (6.1%)	5 (7.6%)		
**Religion**				0.547	χ2
Christian (excluding Pentecostal)	92 (69.7%)	48 (72.7%)	44 (66.7%)		
Pentecostal	31 (23.5%)	15 (22.7%)	16 (24.2%)		
Traditional, other, none	9 (6.8%)	3 (4.6%)	6 (9.1%)		
**Education**				0.382	Fisher’s exact
None or primary	60 (45.8%)	27 (41.5%)	33 (50.0%)		
Secondary or above	71 (58.5%)	38 (58.5%)	33 (50.0%)		
**Not employed**				0.160	χ2
Yes	66 (50.1%)	37 (56.9%)	29 (44.6%)		
No	64 (49.2%)	28 (43.1%)	36 (55.4%)		
**Average household size**	5.9 (±4.5)	6.3 (±5.1)	5.5 (±3.7)	0.317	t-test
**Monthly household income**				0.096	Fisher’s exact
N$ 0–500 (0–59 USD)	67 (58.8%)	37 (71.2%)	30 (48.4%)		
N$501–1000 (59–118 USD)	19 (16.7%)	4 (7.7%)	15 (24.2%)		
N$ 1001–2500 (118–294 USD)	15 (13.2%)	6 (11.5%)	9 (14.5%)		
N$ 2501–5000 (284–588 USD)	8 (7.0%)	3 (5.8%)	5 (8.1%)		
N$ 5000+ (588+ USD)	5 (4.4%)	2 (3.9%)	3 (4.8%)		
**Marital status**				0.156	Fisher’s exact
Married, living together	49 (37.4%)	19 (29.2%)	30 (45.5%)		
Divorced, separated, widowed	17 (13.0%)	10 (15.4%)	7 (10.6%)		
Never married, never lived together	65 (49.6%)	36 (55.4%)	29 (43.9%)		
**Nutrition**					
**Food insecurity** ^a^				0.283	Fisher’s exact
Secure to mild insecurity	81 (61.8%)	44 (66.7%)	37 (56.9%)		
Severe to moderate insecurity	50 (38.2%)	22 (33.3%)	28 (43.1%)		
**Baseline weight (kg)**	57.4 (±10.7)	57.9 (±9.8)	56.8 (±11.5)	0.548	t-test
**Health status**					
**Baseline CD4, cells/mm** ^**3**^	219 (±130)	226 (±129)	213 (±132)	0.590	t-test
**Baseline WHO HIV clinical stage** ^b^				0.020	Fisher’s exact
I or II	96 (76.8%)	54 (85.7%)	42 (67.7%)		
III or IV	29 (23.2%)	9 (14.3%)	20 (32.3%)		
**Baseline ART regimen**				0.131	χ2
Nevirapine-based	105 (79.6%)	56 (84.9%)	49 (74.2%)		
Efavirenz-based	27 (20.5%)	10 (15.2%)	17 (25.8%)		
**Self-reported health rating**				0.294	χ2
Excellent, very good	18 (13.7%)	7 (10.6%)	11 (16.9%)		
Good, fair, poor	113 (86.3%)	59 (89.4%)	546 (83.1%)		
**Depression screen (PHQ-9 score)** ^c^				0.664	Fisher’s exact
Minimal to mild (0–5)	102 (79.7%)	53 (81.5%)	49 (77.8%)		
Moderate to moderately severe (6–20)	26 (20.3%)	12 (18.5%)	14 (22.2%)		
**Health beliefs**					
**How important do you think it is to see a doctor routinely for your HIV care?**				1	Fisher’s exact
Very important	122 (92.4%)	61 (92.4%)	61 (92.4%)		
Somewhat important	7 (5.3%)	3 (4.6%)	4 (6.1%)		
Not important	3 (2.3%)	7 (5.5%)	1 (1.5%)		
**Religious leader told to pray instead of ART**				0.510	χ2
Yes	10 (7.7%)	4 (6.15%)	6 (9.2%)		
No	120 (92.3%)	61 (93.9%)	59 (90.8%)		
**Prefer traditional medicine**				0.119	Fisher’s exact
Yes	4 (3.1%)	0 (0%)	4 (6.2%)		
No	124 (96.9%)	63 (100%)	61 (93.9%)		
**Healthcare access barriers**					
**Difficulty to leave work or home to attend clinic**				0.070	χ2
Easy to little difficult	99 (75.0%)	54 (81.8%)	45 (68.2%)		
Difficult to very difficult	33 (25.0%)	12 (18.2%)	21 (31.8%)		
**Difficulty traveling to clinic**				0.204	χ2
Easy to little difficult	99 (75.6%)	53 (80.3%)	46 (70.8%)		
Difficult to very difficult	32 (24.4%)	13 (19.7%)	19 (29.2%)		
**Average travel time (minutes)**	57.2 (±57.6)	56.4 (±52.6)	58.1 (±62.8)	0.866	t-test
**Average travel cost (N$)**	21.4 (±32.8)	27.9 (±41.22)	15.1 (±20.5)	0.043	t-test
**Walk to clinic**				0.753	χ2
Yes	42 (32.6%)	20 (31.3%)	22 (33.9%)		
No	87 (67.4%)	44 (68.8%)	43 (66.2%)		
**Taxi/bus**				0.349	χ2
Yes	35 (27.1%)	15 (23.4%)	20 (30.8%)		
No	94 (72.9%)	49 (76.6%)	45 (69.2%)		
**Hitchhike**				0.030	χ2
Yes	39 (30.2%)	25 (39.1%)	14 (21.5%)		
No	90 (69.8%)	39 (60.9%)	51 (78.5%)		
**Months lived in baseline town during study period**				0.268	χ2
12	108 (82.4%)	56 (86.2%)	52 (78.8%)		
<12	23 (17.6%)	9 (13.9%)	14 (21.2%)		
**HIV stigma**					
**Family/friends support ARTs**				0.684	Fisher’s exact
Supportive, very supportive	111 (84.7%)	55 (84.6%)	56 (84.9%)		
Neutral	15 (11.5%)	9 (13.9%)	6 (9.1%)		
Opposed, very opposed	5 (3.8%)	1 (1.5%)	4 (6.1%)		
**Stigma score** ^d^				0.006	χ2
0–1	68 (53.5%)	42 (65.6%)	26 (41.3%)		
2–6	59 (46.5%)	22 (34.4%)	37 (58.7%)		
**Non-disclosure of HIV status to certain persons**				0.377	χ2
Yes	38 (29.2%)	17 (25.8%)	21 (32.8%)		
No	92 (70.8%)	49 (74.2%)	43 (67.2%)		

^a^Food insecurity: Household Food Insecurity Access Survey [21].

^b^Baseline WHO HIV clinical stage.

^c^Depression screen: PHQ-9 [22].

^d^Stigma score: Stigma questionnaire developed for study.

## Discussion

With significant progress on the AIDS pandemic by increasing ART coverage, one of the main barriers to viral suppression remains LTFU [3,24,25]. LTFU remains challenging because it is by definition difficult to evaluate which barriers most impact disengagement once patients are lost. Other studies have also reported that LTFU classification shifts occurred after tracing due to underreporting of deaths and silent transfers [2,15,26]. Successful tracing of patients LTFU ranges from 20–100% per one meta-analysis [27]. Our study demonstrated that active tracing yielded a 50% relative drop in LTFU classification (from about 28% to 14%), indicating that half of the patients initially thought to be LTFU were actually engaged in care, deceased, or had transferred to another clinic. This applies to national healthcare implementation in that the post-tracing LTFU rate at 14.3% in fact meets the WHO goal of LTFU <15%, which was not reflected in the pre-tracing rate at 28%. The post-tracing LTFU rate is also lower than the prior WHO-reported LTFU rates in Namibian LTFU at 19.7% in 2008–2009, Southern Africa at 20.1% averaged 2004–2012, and globally at 20% in 2016 [2].

In addition to accurately quantifying LTFU, it is imperative to understand predictors of attrition and design interventions to target those at greatest risk, especially because the highest rate of LTFU often occurs within the first six months of treatment [28]. Unlike some prior studies, this study did not find an association between LTFU and less schooling, being single, lower BMI, lower baseline CD4 counts, and advanced HIV clinical stage [14–19]. However, there is suspected confounding between variables given many were significant in the univariable analysis but not the multivariable model (including language, religion, baseline WHO HIV clinical stage, preference for traditional medicine, difficulty traveling to clinic, hitchhiking, staying in baseline town for the 12 months of the study, and stigma score).

Our study did find that factors associated with LTFU at 12 months included younger age, male sex, difficulty leaving work or home to attend clinic, and initiating ART on an efavirenz-based regimen. Prior literature reported mixed results on age and LTFU: some found that younger patients—adolescents and young adults—are at greater risk for LTFU similar to our study, while others did not find age to be significant [14–17,29]. In applicable contexts, increased LTFU with increasing age may be due to increased obligations with work or family. This may also be due to different generational perceptions of the necessity of ARTs or of HIV stigma. However, HIV stigma was notably not found to be a significant factor in this study. Prior literature is also mixed on male sex as a risk factor: we found that males were at higher risk for attrition like prior multiple studies [15,17,30], while others did not find significance [14,16]. Studies that did also find male sex significant suggest that males may have lower adherence and higher mobility [15]. This may be applicable to Namibia given the prevalence of seasonal work that can be geographically far from one’s baseline ART clinic but would need supplemental data to confirm.

This study found that participants with difficulty leaving work or home to attend ART clinic were over twice as likely to become LTFU. However, other factors related to transportation were surprisingly not significant (including difficulty traveling to clinic, mode of transport, transport cost and distance to clinic). Therefore, it can be theorized that competing obligations at work and home contribute to LTFU more so than transportation barriers in Namibia. This is surprising given transportation is split almost evenly between walking, taxi/bus and hitchhiking (with minimal access to driving) and average travel time to clinic is about one hour. When investigating predictors of successfully tracing those who were initially LTFU, travel cost and hitchhiking were the transportation variables that were significantly associated unsuccessful tracing (i.e. remaining LTFU) in initial hypothesis testing. While regression was not used with this sub-cohort, this is continued evidence that transportation is a barrier to ART adherence in Namibia, even potentially impacting the ability of tracers or other healthcare personnel from reaching patients.

These finding regarding transportation barriers supplement prior literature that has not evaluated competing obligations (whereas they did examine transportation). For example, a systematic review that evaluated the impact of transportation barriers on HIV outcomes in sub-Saharan Africa found that 44% of studies reported a negative impact from transportation barriers, 50% found a null association, and 6% found a paradoxical benefit [31]. On the rare occasion when competing obligations were investigated in prior literature, the method was usually qualitative and cited competing priorities to attending HIV clinic like the inability to leave children unattended and to miss workdays [32,33]. Interestingly, a study in rural Uganda reported that GPS-measured distance but not self-reported transportation barriers were associated with missed HIV clinic visits [34]. This may be an area for further research given self-reported barriers but not GPS-measured distance were evaluated in the current study.

There is a discrepancy across prior studies on whether efavirenz or nevirapine is associated with a greater discontinuation rate [35–38]. This study found that participants on efavirenz-based regimens (14% of participants) were about 2.5 times more likely than those on nevirapine (86%) to become LTFU. This may be due to efavirenz-based regimens being associated with central nervous system (CNS) side effects like dizziness, insomnia, nightmares, and mania [35,37]. Its counterpart—nevirapine—induces what might be considered more minor symptoms such as rash and fever [37]. The WHO recommends transition from efavirenz- to dolutegravir-based regimens, which is currently occurring in 82 low- and middle-income countries, including Namibia. This change will hopefully decrease LTFU based on our study results that efavirenz-based regimens can be associated with higher LTFU, which we hypothesize is related to the aforementioned side effect profiles. For example, dolutegravir may bypass the less tolerable CNS side effects associated with efavirenz that can stem distrust in the medical system and impact patients’ functional ability to return to clinic. Notably, these CNS side effects disproportionately impact patients with psychiatric illness already vulnerable to LTFU [39].

One strength of this study is its prospective nature with tracing to verify LTFU classification. Another strength is assessing barriers to retention in care, as most other studies focus on baseline characteristics. While baseline characteristics are helpful in informing who should be monitored more closely, understanding specific barriers can best inform interventions. One limitation of this study includes its survey-based structure. While this survey allowed consistent implementation in a large number of participants, its moderate number of short-answer questions may not have captured all relevant predictors of LTFU. Additionally, data on dynamic moving in and out of care is not available, as the medical record system did not allow for accurate tracking of patient mobility. Another limitation is the relatively low occurrence of LTFU outcomes (n = 68) compared to the large number of covariates (n = 30), which impacts modelling. Similarly, some associations found significant in other studies but not in this one could be impacted by lack of power.

Overall, interventions addressing the barriers outlined here for patients at highest risk for LTFU may improve retention and viral suppression. Interventions should target younger males and address individuals’ competing obligations, including difficulty leaving their work or home to attend clinic. This is particularly true in the first several months of treatment, given early adherence is a predictor of LTFU, and early retention is associated with VL suppression and better outcomes [20,40,41]. Potential interventions to reduce barriers to getting to clinic and reduce LTFU include multi-month dispending (i.e. provision of between three and six months of pills at a visit), increasing clinic hours, increasing clinic locations, group-based community ART pick-up and distribution, and transitioning to a DTG-based regimen.

## Supporting information

S1 File Baseline questionnaire packet. (PDF)
