# Supplementary material for: Predictors of loss to follow-up from HIV antiretroviral therapy in Namibia
Source: PLoS One. 2022 Apr 14;17(4):e0266438. doi: 10.1371/journal.pone.0266438 (PMC9009635; doi:10.1371/journal.pone.0266438)
Supplement: S1 File — (PDF) [file pone.0266438.s001.pdf]

REPUBLIC OF NAMIBIA

MINISTRY OF HEALTH AND

SOCIAL SERVICES

Namibia Defaulter Tracing Study
Phase 1: Baseline Questionnaire Packet

ENGLISH VERSION

Instructions to Interviewer:

1. Select packet in appropriate language (English, Afrikaans, Oshiwambo or Silosi)
2. Use only one language for each participant
3. Make sure that the packet is complete (15 pages total)
4. Proceed through Baseline Questionnaire Packet in sequential order
5. Write SID Number at top of each page as you go through packet
6. Read ALL questions word-for-word
7. Read ALL answer choices to participant
8. Record ALL answers in English, regardless of language of questionnaire
9. When asking a question with a star (*) use appropriate probe on the approved probe list

INTRODUCTORY PARAGRAPH

Since many people have never been in an interview exactly like this, let me read you a paragraph that tells a little bit about how it works. I am going to read you a set of questions exactly as they are worded so that every respondent in the survey is answering the same questions. You'll be asked to answer two kinds of questions. In some cases, you'll be asked to answer in your own words. For those questions, I will have to write down your answers word for word. In other cases, you will be given a list of answers and asked to choose the one that fits best. If at any time during the interview you are not clear about what is wanted, be sure to ask me.

SID Number: ____ ____ ____ ____

Socioeconomic Status Questionnaire
SES

INSTRUCTIONS TO BE READ TO PARTICIPANT: Please answer each question as accurately and as honestly as you can. All information in this study will be kept confidential. This section asks about family and money.

1.	What is your marital status?	Married or Living Together ☐	Divorced or Separated ☐	Widowed ☐	Never Married and Never Lived Together ☐				
2.	What is your religion?	Lutheran ☐	Anglican ☐	Pentecostal ☐	Catholic ☐	Traditional ☐	None ☐	Other ☐	(IF other) Specify
3.	What is your highest education level attained?	None ☐	Primary (Grades 1-7) ☐	Secondary (Grades 8-12) ☐	Certificate ☐	Higher (Diploma or Bachelor's Degree) ☐	Masters or above ☐		
4.	What is your employment status? *Tick all that apply.*	Full-time ☐	Part-time ☐	Temporary ☐	Not Employed ☐	Welfare ☐			
5.	What is your average monthly household income?#	N$0-500 ☐	N$501-1,000 ☐	N$1001-2,500 ☐	N$2,501-5,000 ☐	N$5,000 and above ☐			
6.	How many people live in your household including you? (physical home)	# People							
7.	What is your current address?	Address							
	7a. Phone number(s) *Probe*: Any others?	Number (s)							
8.	Who is an alternate contact? (in case we cannot find you)	Name/relationship							
	8a. Phone number(s) *Probe*: Any others?	Number (s)							

		Jan	Feb	Mar	Apr	May	Jun	Jul	Aug	Sep	Oct	Nov	Dec
9.	Which months of the year do you live in this city/town/village?# *Tick all that apply.*	☐	☐	☐	☐	☐	☐	☐	☐	☐	☐	☐	☐

	9a. What is your address when you're living outside of this city/town/village?	Address

10. In your current home do you have the following?

Electricity		Solar Electricity		Running Water		Refrigeration		Cooking Gas Stove	
Yes	No	Yes	No	Yes	No	Yes	No	Yes	No
☐	☐	☐	☐	☐	☐	☐	☐	☐	☐

SES (continued)

11.	How difficult is it for you to leave work or home to attend ARV clinic?#	Very Difficult ☐	Difficult ☐	Little Difficult ☐	Easy ☐			
	11a. (IF very difficult or difficult to #11) What are the specific difficulties? *Tick all that apply.*	My employer won't let me miss work ☐	If I miss work, I don't get paid ☐	If I miss work, they might know my HIV status ☐	No one else to care for my children/family members ☐	Other ☐		
	11ai. (IF other) Specify.*	Specify						
12.	How did you travel to the clinic today? *Tick all that apply.*	Walk ☐	Bicycle ☐	Taxi/Bus ☐	Hitchhike ☐	Animal/ Animal Cart ☐	Car/ Motorcycle ☐	Other ☐
	12a. (IF other) Specify.	Specify						
13.	How long does it take you to reach the ARV clinic?	Minutes						
14.	How much does it cost you to reach the ARV clinic?	N$						
	14a. (IF answer to #14 is more than $0) How did you pay to get here?	My own money ☐	Family/friend gave me money ☐	Welfare ☐				
15.	How difficult is it for you to get to the ARV clinic?#	Very Difficult ☐	Difficult ☐	Little Difficult ☐	Easy ☐			

SID Number: ____ ____ ____ ____

HIV Knowledge Questionnaire
<u>HIV KNOWLEDGE</u>

INSTRUCTIONS TO BE READ TO PARTICIPANT: This section asks about your knowledge of HIV.

1.	Do you believe you have HIV?	Yes ☐	No ☐	Don't Know ☐
2.	How reliable do you think the HIV test is?	Very Reliable ☐	Somewhat Reliable ☐	Not Reliable ☐

SID Number: ___ ___ ___ ___

Beliefs about Medicines Questionnaire-ART
BMQ-ART

INSTRUCTIONS TO BE READ TO PARTICIPANT: This section asks what you think about antiretroviral therapy (ARVs).

	**Please indicate the extent to which you agree or disagree with the following statements:**	**Strongly Disagree**	**Disagree**	**Neutral**	**Agree**	**Strongly Agree**
1.	My health in the future will depend on ARVs#	☐	☐	☐	☐	☐
2.	ARVs keep HIV under control#	☐	☐	☐	☐	☐
3.	ARVs will keep me alive#	☐	☐	☐	☐	☐
4.	Taking ARVs will make me less infectious to others.#	☐	☐	☐	☐	☐
5.	Missing ARVs for 2-3 days in a row won't matter in the long run#	☐	☐	☐	☐	☐
6.	I have received enough information about ARVs and I understand them#	☐	☐	☐	☐	☐
7.	Picking up ARVs at the clinic routinely in the future will be difficult#	☐	☐	☐	☐	☐
8.	Taking ARVs everyday as prescribed by my doctor will be difficult#	☐	☐	☐	☐	☐
9.	Using ARVs will be embarrassing#	☐	☐	☐	☐	☐
10.	ARVs will make me feel sick#	☐	☐	☐	☐	☐
11.	I sometimes worry about long-term effects of ARVs#	☐	☐	☐	☐	☐

12.	How do your family/friends feel about your treatment with ARVs?#	Very supportive ☐	Supportive ☐	Neutral ☐	Opposed ☐	Very Opposed ☐
13.	Do you know a family member or close friend who is on ARVs?		Yes ☐		No ☐	

14. What do your family and friends tell you about ARVs?*

Specify

SID Number: ___ ___ ___ ___

Lost to Follow-up Questionnaire
LTFU

INSTRUCTIONS TO BE READ TO PARTICIPANT: This section asks about loss to follow-up. This information will help us understand reasons why patients stop ARV treatment.

1.	How important do you think it is to see a doctor routinely for your HIV care?	Very Important ☐		Somewhat Important ☐		Not Important ☐
2.	How would you rate your experience with the ARV clinic so far?#	Excellent ☐	Very good ☐	Good ☐	Fair ☐	Poor ☐

3. What are the most important things about your experience with the ARV clinic that would make you want to come back?*

Specify

4. What are the most important things about your experience with the ARV clinic that would make you NOT want to come back?*

Specify

5. What is your most important motivation that will keep you coming to the ARV clinic?*

Specify

6. What is the most important barrier/obstacle for you to keep coming to the ARV clinic?*

Specify

6a. How will you overcome these barriers/obstacles?*

Specify

SID Number: ___ ___ ___ ___

Alternative Healing Questionnaire
ALTER

INSTRUCTIONS TO BE READ TO PARTICIPANT: This section asks about alternative healing.

1.	Has your religious leader told you not to take ARVs or told you to pray instead of taking ARVs?		Yes ☐		No ☐	
2.	Do you believe that God can heal HIV through prayer?		Yes ☐	No ☐		Don't know ☐
	2a. (If YES to #2) Do you believe that taking ARVs shows a lack of faith in God's ability to heal your HIV?		Yes ☐	No ☐		Don't know ☐
	2b. (If YES to #2) How do you know if God has healed you of HIV?	Get retested ☐	Stop ARVs and see if sick ☐	Religious leader tells me ☐	Don't know ☐	Other ☐
	2bi. (IF other) Specify.*	Specify				
3.	Do you regularly see a traditional healer?		Yes ☐		No ☐	
4.	Do you prefer traditional medicine to western medicine for treating HIV?		Yes ☐		No ☐	
5.	Is traditional medicine more affordable than western medicine?		Yes ☐	No ☐		Don't know ☐
6.	Do you live closer to a traditional healer than the ARV clinic?		Yes ☐	No ☐		Don't know ☐
7.	Does traditional medicine work faster than ARVs?		Yes ☐	No ☐		Don't know ☐
8.	Can traditional medicine cure HIV?		Yes ☐	No ☐		Don't know ☐
9.	Does traditional medicine have fewer bad effects than ARVs?		Yes ☐	No ☐		Don't know ☐

SID Number: ___ ___ ___ ___

Physical Health Questionnaire
PHYSICAL HEALTH

INSTRUCTIONS TO BE READ TO PARTICIPANT: This section asks about how healthy you feel.

(For Q#1-4) DURING THE PAST THREE MONTHS:

1. Overall, how would you rate your physical health?#

Excellent	Very good	Good	Fair	Poor
☐	☐	☐	☐	☐

2. How much did physical health problems keep you from doing your usual work, school or social activities?

Not at all	Little	Quite a lot	Could not do activities
☐	☐	☐	☐

3. How much physical energy did you have?

None	Little	Normal amount	Quite a lot
☐	☐	☐	☐

4. How much bodily pain have you had?

None	Little	Moderate amount	Quite a lot
☐	☐	☐	☐

5. If you feel well right now, do you need to take ARVs?

Yes	No	Don't Know
☐	☐	☐

6. Can a person who has HIV look or feel healthy?

Yes	No	Don't Know
☐	☐	☐

7. If you are taking ARVs and feel better do you need to keep taking them?

Yes	No	Don't Know
☐	☐	☐

8. If you are taking ARVs and not feeling better right away, are they working?

Yes	No	Don't Know
☐	☐	☐

SID Number: ___ ___ ___ ___

Patient Health Questionnaire
PHQ-9

INSTRUCTIONS TO BE READ TO PARTICIPANT: This section asks you how you feel emotionally.

	Over the last 2 weeks, how often have you been bothered by any of the following problems?	**Not at all**	**Several days**	**More than half the days**	**Nearly every day**
1.	Little interest or pleasure in doing things#	☐	☐	☐	☐
2.	Feeling down, depressed, or hopeless#	☐	☐	☐	☐
3.	Have poor sleep; or sleep too much#	☐	☐	☐	☐
4.	Feeling tired or having little energy#	☐	☐	☐	☐
5.	Not feel like eating or your appetite was poor; or overeating#	☐	☐	☐	☐
6.	Think your life had been a failure or let your family down#	☐	☐	☐	☐
7.	Have trouble keeping your mind on what you were doing#	☐	☐	☐	☐
8.	Moving or speaking slowly so people notice or restless#	☐	☐	☐	☐
9.	Thoughts that you would be better off dead or of hurting yourself#	☐	☐	☐	☐

SID Number: ___ ___ ___ ___

Social Support Questionnaire
SUPPORT

INSTRUCTIONS FOR INTERVIEWER: This section asks about social support.

1.	If I were sick and needed someone to take me to a doctor I would have trouble finding someone.#	Always True ☐	Mostly True ☐	Mostly False ☐	Always False ☐
2.	I feel that there is no one I can share my most private concerns and fears.#	Always True ☐	Mostly True ☐	Mostly False ☐	Always False ☐
3.	I feel no strong emotional bond with another person.#	Always True ☐	Mostly True ☐	Mostly False ☐	Always False ☐

SID Number: ___ ___ ___ ___

Household Food Insecurity Access Survey
HFIAS

INSTRUCTIONS TO BE READ TO PARTICIPANT: This section asks for your level of food security.

1.	In the past four weeks, did you worry that your household would not have enough food?		Yes ☐	No (skip to Q2) ☐
	1a. How often did this happen?#	Rarely (once or twice in the past four weeks) ☐	Sometimes (three to ten times in the past four weeks) ☐	Often (more than ten times in the past four weeks) ☐
2.	In the past four weeks, were you or any household member not able to eat the kinds of foods you preferred because of a lack of resources?		Yes ☐	No (skip to Q3) ☐
	2a. How often did this happen?#	Rarely (once or twice in the past four weeks) ☐	Sometimes (three to ten times in the past four weeks) ☐	Often (more than ten times in the past four weeks) ☐
3.	In the past four weeks, did you or any household member have to eat a limited variety of foods due to a lack of resources?		Yes ☐	No (skip to Q4) ☐
	3a. How often did this happen?#	Rarely (once or twice in the past four weeks) ☐	Sometimes (three to ten times in the past four weeks) ☐	Often (more than ten times in the past four weeks) ☐
4.	In the past four weeks, did you or any household member have to eat some foods that you really did not want to eat because of a lack of resources to obtain other types of food?		Yes ☐	No (skip to Q5) ☐
	4a. How often did this happen?#	Rarely (once or twice in the past four weeks) ☐	Sometimes (three to ten times in the past four weeks) ☐	Often (more than ten times in the past four weeks) ☐
5.	In the past four weeks, did you or any household member have to eat a smaller meal than you felt you needed because there was not enough food?		Yes ☐	No (skip to Q6) ☐
	5a. How often did this happen?#	Rarely (once or twice in the past four weeks) ☐	Sometimes (three to ten times in the past four weeks) ☐	Often (more than ten times in the past four weeks) ☐
6.	In the past four weeks, did you or any household member have to eat fewer meals in a day because there was not enough food?		Yes ☐	No (skip to Q7) ☐
	6a. How often did this happen?#	Rarely (once or twice in the past four weeks) ☐	Sometimes (three to ten times in the past four weeks) ☐	Often (more than ten times in the past four weeks) ☐

<u>HFIAS</u> (continued)

7.	In the past four weeks, was there ever no food to eat of any kind in your household because of lack of resources to get food?		Yes ☐	No (skip to Q8) ☐
	7a. How often did this happen?#	Rarely (once or twice in the past four weeks) ☐	Sometimes (three to ten times in the past four weeks) ☐	Often (more than ten times in the past four weeks) ☐
8.	In the past four weeks, did you or any household member go to sleep at night hungry because there was not enough food?		Yes ☐	No (skip to Q9) ☐
	8a. How often did this happen?#	Rarely (once or twice in the past four weeks) ☐	Sometimes (three to ten times in the past four weeks) ☐	Often (more than ten times in the past four weeks) ☐
9.	In the past four weeks, did you or any household member go a whole day and night without eating anything because there was not enough food?		Yes ☐	No (skip to next section) ☐
	9a. How often did this happen?#	Rarely (once or twice in the past four weeks) ☐	Sometimes (three to ten times in the past four weeks) ☐	Often (more than ten times in the past four weeks) ☐

SID Number: ___ ___ ___ ___

Risks Questionnaire
RISKS

INSTRUCTIONS TO BE READ TO PARTICIPANT: This section asks about drugs, alcohol and other risky behaviors. Please answer each question as accurately and as honestly as you can. Again you are reassured that all information in this study will be kept confidential and will not affect your medical care.

1.	In the past three months how often did you drink alcohol? (including purchased alcohol and homebrew)#	0 times/month ☐	Once or less/week ☐	Few times/week ☐	Daily or more than daily ☐
1a.	(IF more than 0 times/month to #1) How concerned are you about taking ARVs and alcohol at the same time?#		Not concerned ☐	Concerned ☐	Very concerned ☐
2.	In the past three months how often did you use recreational drugs? (i.e. dagga, glue, mandrax, cocaine, rock, drugs with syringe)#	0 times/month ☐	Once or less/week ☐	Few times/week ☐	Daily or more than daily ☐
2a.	(If more than 0 times/month to #2) How concerned are you about taking ARVs and recreational drugs at the same time?#		Not concerned ☐	Concerned ☐	Very concerned ☐
3.	How concerned are you about taking ARVs and having sex at the same time?#		Not concerned ☐	Concerned ☐	Very concerned ☐

Stigma Questionnaire
STIGMA

INSTRUCTIONS FOR INTERVIEWER: This section asks about HIV stigma and discrimination experiences.

1.	It is difficult to tell people about my HIV infection.	Agree ☐		Disagree ☐
2.	Being HIV positive makes me feel dirty.	Agree ☐		Disagree ☐
3.	I feel guilty that I am HIV positive.	Agree ☐		Disagree ☐
4.	I am ashamed that I am HIV positive.	Agree ☐		Disagree ☐
5.	I sometimes feel worthless because I am HIV positive.	Agree ☐		Disagree ☐
6.	I hide my HIV status from others.	Agree ☐		Disagree ☐
7.	Have you been treated differently since you disclosed your HIV status to friends and family?	Yes ☐	No ☐	Never disclosed ☐
8.	Are there people you have not told that you are HIV positive out of fear of negative consequences?	Yes ☐		No ☐

THIS IS THE END OF THE BASELINE QUESTIONNAIRE PACKET
